# Distinct Requirements for Tail-Anchored Membrane Protein Biogenesis in Escherichia coli

**DOI:** 10.1128/mBio.01580-19

**Published:** 2019-10-15

**Authors:** Markus Peschke, Mélanie Le Goff, Gregory M. Koningstein, Norbert O. Vischer, Abbi Abdel-Rehim, Stephen High, Peter van Ulsen, Joen Luirink

**Affiliations:** a The Amsterdam Institute of Molecules, Medicines and Systems, VU University Amsterdam, Amsterdam, The Netherlands; b Swammerdam Institute for Life Sciences, Department of Bacterial Cell Biology and Physiology, University of Amsterdam, Amsterdam, The Netherlands; c Division of Molecular and Cellular Function, School of Biological Sciences, Faculty of Biology, Medicine and Health, University of Manchester, Manchester, United Kingdom; Stony Brook University; Harvard Medical School

**Keywords:** *Escherichia coli*, hydrophobicity, membrane proteins, membrane targeting, tail-anchored

## Abstract

Tail-anchored membrane proteins (TAMPs) are a distinct subset of inner membrane proteins (IMPs) characterized by a single C-terminal transmembrane domain (TMD) that is responsible for both targeting and anchoring. Little is known about the routing of TAMPs in bacteria. Here, we have investigated the role of TMD hydrophobicity in tail-anchor function in Escherichia coli and its influence on the choice of targeting/insertion pathway. We created a set of synthetic, fluorescent TAMPs that vary in the hydrophobicity of their TMDs and corresponding control polypeptides that are extended at their C terminus to create regular type II IMPs. Surprisingly, we observed that TAMPs have a much lower TMD hydrophobicity threshold for efficient targeting and membrane insertion than their type II counterparts. Using strains conditional for the expression of known membrane-targeting and insertion factors, we show that TAMPs with strongly hydrophobic TMDs require the signal recognition particle (SRP) for targeting. Neither the SecYEG translocon nor YidC appears to be essential for the membrane insertion of any of the TAMPs studied. In contrast, corresponding type II IMPs with a TMD of sufficient hydrophobicity to promote membrane insertion followed an SRP- and SecYEG translocon-dependent pathway. Together, these data indicate that the capacity of a TMD to promote the biogenesis of E. coli IMPs is strongly dependent upon the polypeptide context in which it is presented.

## Abstract (precis)

A subset of membrane proteins is targeted to and inserted into the membrane via a hydrophobic transmembrane domain (TMD) that is positioned at the very C terminus of the protein. The biogenesis of these so-called tail-anchored proteins (TAMPs) has been studied in detail in eukaryotic cells. Various partly redundant pathways were identified, the choice for which depends in part on the hydrophobicity of the TMD. Much less is known about bacterial TAMPs. The significance of our research is in identifying the role of TMD hydrophobicity in the routing of E. coli TAMPs. Our data suggest that both the nature of the TMD and its role in routing can be very different for TAMPs versus “regular” membrane proteins. Elucidating these position-specific effects of TMDs will increase our understanding of how prokaryotic cells face the challenge of producing a wide variety of membrane proteins.

## INTRODUCTION

Roughly 30% of a cellular proteome is made up of membrane proteins (1) that are synthesized in the cytoplasm and then have to be targeted to and inserted into their host membranes. Since the hydrophobic transmembrane domains (TMDs) of integral membrane proteins are prone to aggregation in the aqueous cytoplasm, it is critical to shield the TMDs prior to insertion into the lipid bilayer. The majority of integral membrane proteins are targeted to the inner membrane of prokaryotes or the membrane of the eukaryotic endoplasmic reticulum (ER) by the conserved signal recognition particle (SRP) in a cotranslational manner (2, 3). During translation, the TMD of the nascent protein is contacted by the SRP and targeted to the membrane, where it can be transferred into the Sec translocon and subsequently partition laterally into the plasma membrane of prokaryotes or the ER membrane of eukaryotes, respectively. Intriguingly, a small subset of integral membrane proteins depend on a single C-terminal TMD for targeting to and anchoring into the membrane (4). The TMD of these so-called tail-anchored membrane proteins (TAMPs) can only engage targeting factors in the cytoplasm outside the ribosomal exit tunnel when translation is completed, i.e., posttranslationally.

Since TAMPs play crucial physiological roles in organisms of all kingdoms of life, extensive genetic, biochemical, and structural studies have been dedicated to elucidating the mechanisms by which TAMPs reach their target membranes (5). Although a few eukaryotic TAMPs are capable of unassisted membrane insertion (6), the majority follow one of up to five, partly redundant, chaperone-mediated pathways, the choice for which depends in part on the hydrophobicity of the TMD. Hence, although the conserved GET (Guided Entry of TA proteins)/TRC40 pathway is responsible for the majority of TAMP biogenesis in yeast and mammals, respectively, other routes are mediated by the chaperone couple Hsc70/Hsp40, SRP acting in an atypical posttranslational fashion, the SND (SRP-independent targeting) pathway, and the recently discovered ER membrane protein complex (EMC) (5, 7–9).

In prokaryotes, much less is known about the biogenesis of TAMPs (10). SciP was described as a TAMP in Escherichia coli, although in practice it may contain cryptic hydrophobic sequences in the N-terminal region of the protein that can mediate cotranslational targeting via SRP (11). We have recently identified DjlC and Flk as bona fide E. coli TAMPs, the TMDs of which are required and sufficient for membrane targeting and insertion (12). The SRP, its membrane receptor FtsY, the membrane insertase YidC, and the cytoplasmic chaperone DnaK appeared required for optimal targeting and membrane insertion of these proteins (12). Additionally, results indicated that the comparatively more hydrophobic TMD of Flk imposed a stronger dependency on SRP than that seen with the less hydrophobic TMD of DjlC. Possibly, TMD hydrophobicity can in part dictate the preferred targeting and insertion pathway for E. coli TAMPs as it does in eukaryotes (5, 7, 9).

**FIG 1:**
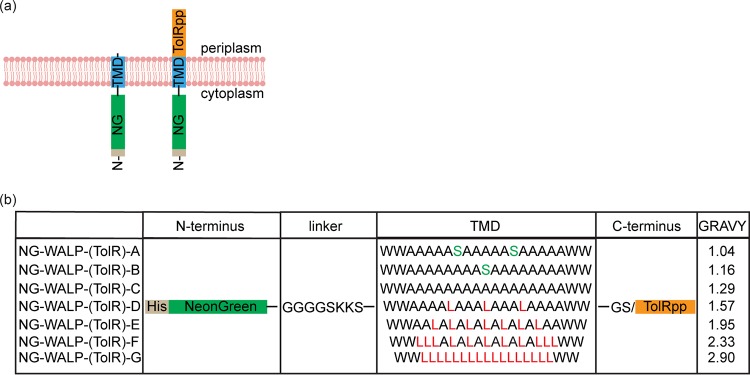
Synthetic TAMPs and type II IMPs. (a) Schematic representation of the topology of the NG-WALP and NG-WALP-TolR constructs. (b) The constructs consist of N-terminal His-tagged mNeonGreen, followed by a flexible linker and a TMD with various degrees of hydrophobicity, as indicated by GRAVY scores. NG-WALP constructs contain the two residues GS and a C-terminal tail, whereas NG-WALKP-TolR constructs are extended with the periplasmic domain of TolR to create a (non-tail-anchored) type II IMP.

To systematically investigate the influence of TMD hydrophobicity on membrane targeting and insertion, we created synthetic TAMPs consisting of the fluorescent reporter mNeonGreen (NG) (13) fused to artificial TMDs composed of different WALPs. WALPs are peptides that consist solely of alanine (A), leucine (L), and tryptophan (W) residues and can serve as synthetic TMDs of various hydrophobicities (14). Surprisingly, we found that the TMD hydrophobicity threshold required to mediate the targeting and membrane insertion of TAMPs is substantially lower than that needed for regular type II inner membrane proteins (IMPs) with the same topology. These type II IMPs have the same N-in C-out orientation as TAMPs but are distinguished by a longer C-terminal domain that enables cotranslational interactions with both cytosolic targeting and membrane insertase factors (Fig. 1). Furthermore, we find that while a moderately hydrophobic TMD appears to localize TAMPs via an unassisted mechanism, TAMPs with a strongly hydrophobic TMD require SRP to act via a mechanism that is posttranslational. The requirement for membrane insertases also differed dramatically between TAMPs and corresponding type II IMPs. Whereas depletion of either YidC or the Sec translocon had little impact on TAMP localization, all membrane-localized type II IMPs strictly required the Sec translocon (but not YidC) for membrane insertion.

## RESULTS

### Localization of synthetic tail-anchored and corresponding type II IMPs.

To investigate the potential influence of TMD hydrophobicity on membrane targeting and insertion, we engineered a set of seven synthetic TAMPs (here designated NG-WALPs) with various degrees of TMD hydrophobicity. The fluorescent reporter protein mNeonGreen (13) was fused to a linker of eight residues followed by a synthetic WALP of 21 residues and a short C-terminal tail of two hydrophilic residues (Fig. 1). The linker consisted of glycine and serine residues for conformational flexibility of N-terminal fusions and two lysine residues to maintain the N-terminal region in the cytoplasm according to the positive inside rule (1, 15). In the least hydrophobic WALPs, we also introduced one or two serine residues in order to achieve a variation in grand average hydropathy (GRAVY) from 1.04 to 2.90 (Fig. 1). This range was chosen as it resembles the range of TMD hydrophobicities found in natural TAMPs like the eukaryotic cytochrome *b*_5_ (Cytb5) (1.03) and the bacterial Flk (2.83).

To investigate whether the hydrophobicity of the TMD has a specific impact on the efficiency and mechanism of TAMP targeting/insertion, compared to that of regular type II IMPs, we made a second set of synthetic membrane proteins. For these proteins (here designated NG-WALP-TolRs), the extremely short periplasmic C terminus of each TAMP was replaced by the 102-residue periplasmic domain of the type II IMP TolR (Fig. 1).

**FIG 2:**
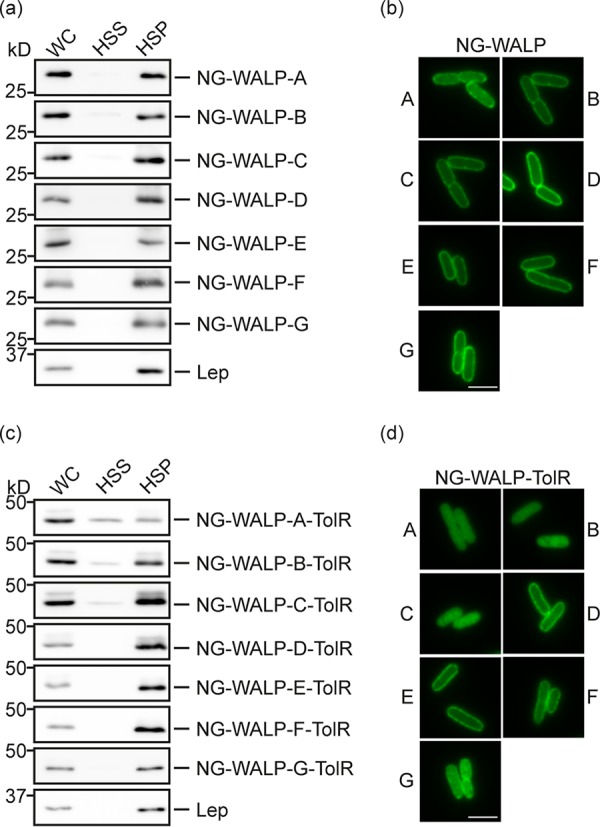
Subcellular localization of NG-WALP and NG-WALP-TolR constructs. E. coli MC4100 cells expressing His-tagged NG-WALP and NG-WALP-TolR constructs were lysed and subjected to ultracentrifugation or fixed for fluorescence microscopy. (a and c) Whole-cell (WC), high-speed supernatant (HSS), and high-speed pellet (HSP) fractions were analyzed by Western blotting using anti-His serum. Lep was detected by specific antibodies as an inner membrane protein control. (b and d) A portion of the cells was fixed with formaldehyde and analyzed by fluorescence microscopy. Bars, 3 μm.

We first examined the effect of TMD hydrophobicity on TAMP localization by expressing the NG-WALPs in wild-type E. coli followed by cell fractionation, membrane extraction, and fluorescence microscopy. Strikingly, all seven fusion proteins were detected exclusively in the high-speed pellet consistent with their localization in/at the inner membrane as observed with the control endogenous integral membrane protein leader peptidase (Lep) (Fig. 2a). To investigate this membrane association in more detail, membranes were either salt washed with phosphate-buffered saline (PBS) or treated with detergent (2% *N*-dodecyl-β-d-maltoside [DDM]). PBS extracted a minor fraction of the least hydrophobic model TAMP, NG-WALP-A, but little else (see Fig. S1a in the supplemental material). In contrast, 2% DDM resulted in complete solubilization and extraction of all NG-WALPs and Lep (Fig. S1a). These data indicate that all NG-WALPs are integral membrane proteins except for a small fraction of the least hydrophobic one, NG-WALP-A, which appears to be associated with but not fully inserted into the E. coli inner membrane.

10.1128/mBio.01580-19.1 FIG S1 Membrane insertion analyses of NG-WALP and NG-WALP-TolR constructs. (a and c) Crude membranes from E. coli MC4100 expressing NG-WALP and NG-WALP-TolR constructs acquired as described in the legend to Fig. 2 were extracted with PBS or 2% DDM. Subsequently, samples were centrifuged and supernatant (S) and pellet (P) fractions were analyzed by Western blotting using anti-His and anti-Lep sera. Lep served as inner membrane protein control. (b and d) E. coli MC4100 cells expressing NG-WALP and NG-WALP-TolR constructs were converted to spheroplasts and treated with PBS, ProtK alone, or ProtK and Triton. Samples were analyzed by Western blotting using anti-His, antiopsin, and anti-TF sera. TF was used as a cytoplasmic protein control. Truncated forms of NG-WALP-TolR-E/F of ∼30 kDa that also appear in PBS-treated samples might indicate intrinsic cleavage of these constructs. Download FIG S1, TIF file, 1.0 MB. Copyright © 2019 Peschke et al. 2019 Peschke et al. This content is distributed under the terms of the Creative Commons Attribution 4.0 International license.

Fluorescence microscopy analysis supported these fractionation data, and we observed a disperse circumferential labeling at the cell envelope for all NG-WALPs (Fig. 2b). To test for authentic membrane insertion including full translocation of the short C-terminal tail, we added a short 13-residue extension in the form of an opsin tag to the C termini of NG-WALP-B/C/G. This tag is derived from the N-terminal domain of bovine opsin and known to be compatible with translocation across the bacterial inner membrane (12). These derivatives were used to investigate the translocation of the C-terminal tail across the inner membrane using a protease protection assay. E. coli MC4100 cells expressing opsin-tagged NG-WALP-B/C/G were converted to spheroplasts and incubated with proteinase K (ProtK). All three TAMPs were detected by their N-terminal His tag and C-terminal opsin tag in control PBS-treated spheroplasts (Fig. S1b). Upon treatment with ProtK, the N-terminal His tag remained intact for all three proteins, whereas the opsin tag was barely detectable (Fig. S1b), indicating that the C-terminal tail was successfully translocated across the inner membrane and thus susceptible to ProtK. To confirm the integrity of the inner membrane in the spheroplasts, we monitored the cytoplasmic control protein trigger factor (TF), which remained unaffected by ProtK unless the spheroplasts were treated with Triton X-100 (Fig. S1b). Taken together, the results indicate that the NG-WALPs are authentic integral membrane proteins and hence bona fide TAMPs.

Having established that an artificial TMD of comparatively low hydrophobicity is sufficient for the stable membrane anchoring of the model TAMPs, we next studied the significance of the proximity of the same TMDs to the C terminus of the protein. To this end, we determined the subcellular location of the seven corresponding type II IMPs (NG-WALP-TolRs) by fractionation. Proteins with more hydrophobic TMDs (NG-WALP-TolR-D/E/F/G) were exclusively detected in the high-speed pellet, as was our control integral membrane protein, Lep (Fig. 2c). In contrast, a fraction of the proteins with less hydrophobic TMDs, NG-WALP-TolR-A/B/C, was consistently recovered in the high-speed supernatant, suggesting a more peripheral membrane association. Furthermore, NG-WALP-TolR-A and, to a lesser extent, WALP-TolR-B/C could be released from the membrane using PBS (Fig. S1c). Similarly to Lep, membrane-associated NG-WALP-TolR-A to -F were efficiently extracted from the membrane pellet using 2% DDM, and only small amounts remained in the pellet fraction, perhaps reflecting insoluble protein aggregates (Fig. S1c). When the subcellular localization of the proteins was analyzed by fluorescence microscopy, NG-WALP-TolR-A/B/C showed a diffuse signal throughout the cell in contrast to the disperse circumferential labeling of NG-WALP-TolR-D/E/F (Fig. 2d), further underlining the differences in their membrane association. Surprisingly, this “halo” formation was barely visible for NG-WALP-TolR-G, which contains the most hydrophobic TMD of our series (Fig. 2d). Interestingly, NG-WALP-TolR-G was also the only model type II IMP that was inefficiently solubilized by 2% DDM (Fig. S1c), and we conclude that this protein is most likely aggregation prone and hence inefficiently inserted into the membrane.

To determine if the efficiently membrane-localized NG-WALP-TolR-D/E/F proteins are truly membrane inserted, we analyzed their accessibility to ProtK in spheroplasts. Control PBS-treated samples showed all three proteins in their full-length form detected by their N-terminal His tag (Fig. S1d). Upon treatment with ProtK, the full-length forms had almost completely disappeared and truncated forms of ∼31 kDa accumulated consistent with the size of the NG-WALP region alone, indicating that their C-terminal TolR domain had passed across the inner membrane and was digested by ProtK. Since NG-WALP-TolR-B showed no obvious membrane localization by fluorescence microscopy, we used it as a negative control and confirmed that the TolR domain is located in the cytoplasm where both it and cytoplasmic control TF are protected from ProtK (Fig. S1d).

Together, the fractionation, protease accessibility, and fluorescence microscopy data suggest that while NG-WALP-TolR-D/E/F proteins are correctly inserted type II IMPs, the less hydrophobic NG-WALP-TolR-A/B/C proteins remain in the cytoplasm. This is quite different from their tail-anchored counterparts, where NG-WALP-B/C appear to be fully membrane inserted and even NG-WALP-A is tightly membrane bound. The predominant cofractionation of NG-WALP-TolR-B/C proteins with the membranes may be an artifact of the procedure or reflect the formation of small aggregates as observed earlier (12). In any case, we deemed whole-cell fluorescence microscopy the most reliable way to determine the localization of our synthetic membrane proteins and used it in follow-up experiments to determine the requirements for membrane insertion using strains conditional for the expression of targeting and insertion factors (see below).

### DnaK prevents protein aggregation during membrane targeting.

**FIG 3:**
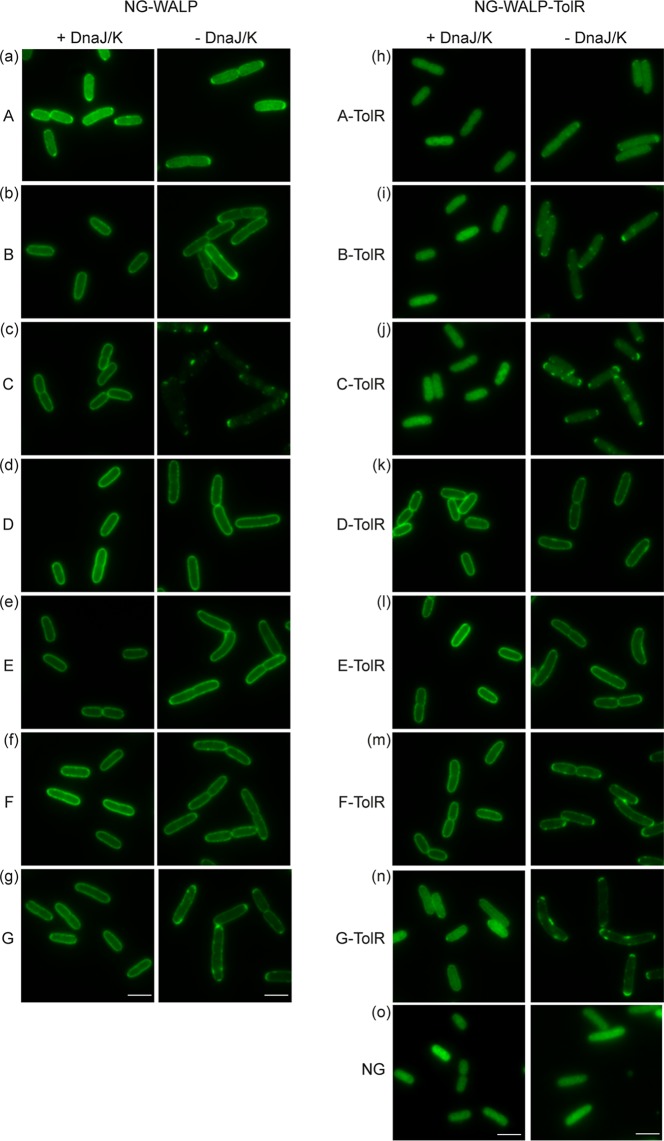
Role of DnaJ/K in the targeting of NG-WALP and NG-WALP-TolR constructs. (a to o) NG-WALP and NG-WALP-TolR constructs were expressed in a DnaJ/K-knockout strain and its isogenic wild-type MC4100. Cells were fixed with formaldehyde and analyzed by fluorescence microscopy. mNeonGreen (NG) was used as a cytoplasmic control protein. Bars, 3 μm.

Having previously established that the cytoplasmic chaperone pair DnaJ/K is involved in the targeting of E. coli TAMPs DjlC and Flk (12), we investigated how changes in TMD hydrophobicity affect the role of DnaJ/K in the membrane insertion of our model TAMP and type II IMPs. Since DnaJ/K is not essential at lower temperatures (16), we could test its role by expressing the model proteins in a DnaJ/K-knockout strain and its isogenic wild-type (MC4100) control. As before (cf. Fig. 2b), all seven NG-WALP constructs showed halo formation, indicating membrane localization in the presence of DnaJ/K (Fig. 3a to g). In the absence of DnaJ/K, NG-WALP-A/B still formed a halo at the cell envelope but in addition also showed small fluorescent dots at the cell poles for NG-WALP-B (Fig. 3b), which might indicate minor aggregate formation. In contrast, NG-WALP-C showed a severe localization defect in the absence of DnaJ/K, with no halo apparent and punctate signals throughout the cell and accumulated at the cell poles (Fig. 3c, -DnaJ/K). NG-WALP-D/E/F remained unaffected in the knockout strain and showed membrane localization similar to the wild-type strain (Fig. 3d to f). The model TAMP with the most hydrophobic TMD, NG-WALP-G, showed membrane localization combined with strong punctate signals at the cell poles, suggesting that substantial aggregation had occurred (Fig. 3g). We additionally analyzed the fluorescence distribution by creating cross-sectional profiles of the cells and assessing membrane localization based on the center/border ratio of the fluorescent signal. This semiquantitative analysis confirmed that there is little to no effect on the membrane localization of NG-WALP-A/B/D/E/F/G in the absence of DnaJ/K in contrast to the severely affected NG-WALP-C (Fig. S3a to g and Fig. S7a). These results suggest that DnaJ/K is only strictly required for the membrane localization of NG-WALP-C, whereas the other constructs are still targeted to the membrane in their absence. Hence, a simple correlation between the hydrophobicity of the TMD in the tail-anchored constructs and the requirement of DnaJ/K for localization is not evident.

10.1128/mBio.01580-19.3 FIG S3 Fluorescence profiles MC4100 and MC4100*ΔdnaJ/K* cells (a to n). For Fig. S3 to S6, software ImageJ, plugin ObjectJ, Coli-Inspector, and CrossProfilesMacro1.0 were used. Cross profiles of cells (*n*) were used to calculate the average fluorescence profile perpendicular to the cell axis of MC4100 (+ DnaJ/K) and MC4100Δ*dnaJ/K* (-DnaJ/K) cells expressing NG-WALP and NG-WALP-TolR constructs. Cell count was determined automatically by the software, and variation stems from clumping of cells leading to different cell densities on the agarose pad. Download FIG S3, TIF file, 2.7 MB. Copyright © 2019 Peschke et al. 2019 Peschke et al. This content is distributed under the terms of the Creative Commons Attribution 4.0 International license.

10.1128/mBio.01580-19.7 FIG S7 Center/border ratio of the fluorescence signal (a to h). Center/border ratios of the fluorescence signal were calculated from cross profiles (see Fig. S3 to S6). Black lines indicate the median of the center/border ratios automatically calculated by software GraphPad Prism8. NG was used as a cytoplasmic control protein. Download FIG S7, TIF file, 1.9 MB. Copyright © 2019 Peschke et al. 2019 Peschke et al. This content is distributed under the terms of the Creative Commons Attribution 4.0 International license.

In the case of the type II IMPs, the membrane localization of NG-WALP-TolR-D and -E is independent of DnaJ/K, while NG-WALP-TolR-F displayed some accumulation of spots at the cell poles (Fig. 3k to i). The mostly cytoplasmic constructs (A/B/C/G) showed spots to various degrees that seem to correlate with TMD hydrophobicity (G > C > B > A). For NG-WALP-G, the center/border ratio of the fluorescent signal decreased in the absence of DnaK/J (Fig. S7b), which may be related to the relocation of disperse cytosolic protein to mostly polar spots. As expected, the diffuse cytoplasmic localization of NG was not affected in the absence of DnaJ/K (Fig. 3o), showing that the observed effects are independent of the reporter protein. The absence of DnaK in the DnaJ/K-knockout strain was confirmed by Western blotting (Fig. S2a).

10.1128/mBio.01580-19.2 FIG S2 Control blotting assays for depletion and deletion strains. NG-WALP and NG-WALP-TolR constructs were expressed in a DnaJ/K-knockout strain, its isogenic wild-type strain, and strains conditional for the expression of Ffh, YidC, and SecE (Fig. 3 and 6). (a) Whole-cell samples of E. coli MC4100*ΔdnaJK* (−) and MC4100 (+) were analyzed by Western blotting using anti-DnaK serum. (b to d) Levels of Ffh and YidC in strains HDB51, MK6s, and CM124 were checked under depleted (−) and nondepleted (+) conditions using anti-Ffh and anti-YidC sera. For strains HDB51 and MK6s, successful processing of SurA was used as an overdepletion control. Control samples (Ctrl) showing accumulation of unprocessed SurA were loaded for HDB51 and MK6s samples. The pre-SurA control sample was obtained from whole cells of the temperature-sensitive SecA strain MM52 grown under nonpermissive conditions. For CM124, accumulation of pre-SurA served as a control for successful depletion of SecE. Download FIG S2, TIF file, 1.6 MB. Copyright © 2019 Peschke et al. 2019 Peschke et al. This content is distributed under the terms of the Creative Commons Attribution 4.0 International license.

Combined, the data suggest that DnaJ/K is not strictly required for targeting of the membrane-localized TAMP and type II IMP constructs except for NG-WALP-C. Although DnaJ/K does not seem to be involved in the targeting of most constructs, the results do indicate that it prevents the aggregation of membrane proteins that mislocalize to the cytosol.

### TAMPs with very hydrophobic TMDs are strictly dependent on SRP for targeting.

We previously obtained evidence that the E. coli TAMPs Flk and DjlC require SRP for membrane targeting (12). Interestingly, the protein with the more hydrophobic TMD (Flk) showed more severe localization defects. This might indicate that, similarly to eukaryotic TAMPs (7, 17), the bacterial TAMPs with more hydrophobic TMDs can utilize SRP for membrane targeting. To investigate the role of the SRP in the targeting of model TAMPs and type II IMPs with TMDs of various hydrophobicities, we expressed the two sets of constructs in HDB51 (18). In this strain, the expression of Ffh, the protein subunit of SRP, is controlled by an l-arabinose-inducible promoter. As observed before, depletion of the SRP led to modest cell filamentation (19). The absence of Ffh under depleted conditions was confirmed by Western blotting (Fig. S2b). To rule out any secondary effects on other targeting and insertion machineries caused by a prolonged absence of SRP (“overdepletion”), we monitored the accumulation of unprocessed SurA (pre-SurA), an SRP-independent periplasmic protein. No accumulation of pre-SurA occurred under the experimental conditions used, indicating that overdepletion had not occurred (Fig. S2b, cf. control sample).

**FIG 4:**
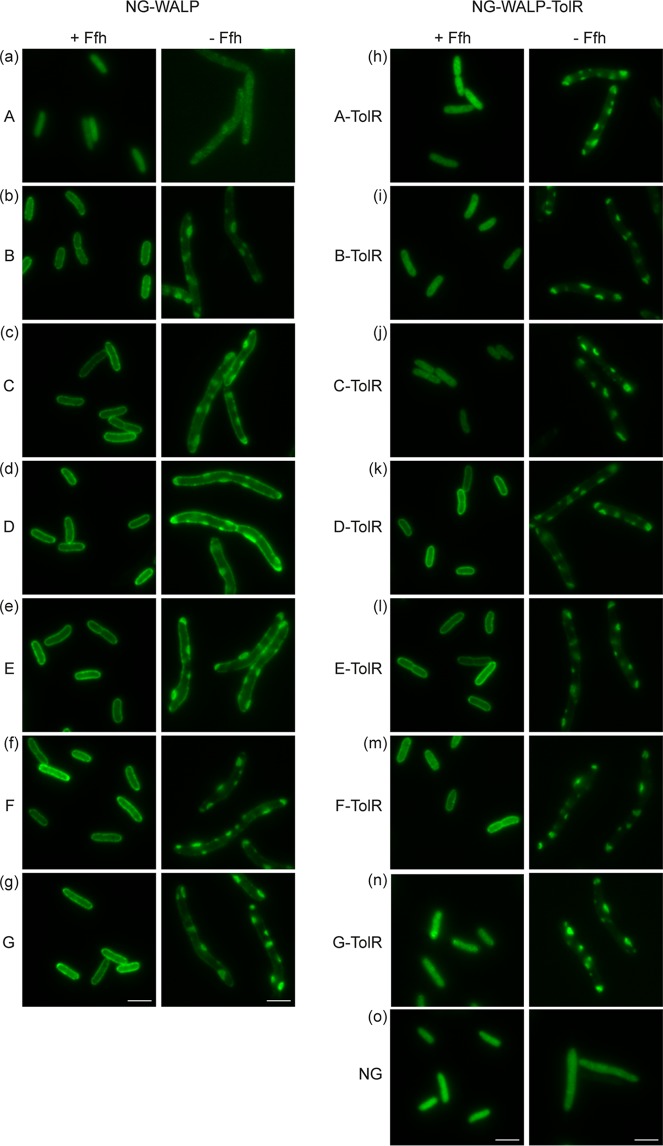
Role of SRP in the targeting of NG-WALP and NG-WALP-TolR constructs. (a to o) NG-WALP and NG-WALP-TolR constructs were expressed under depleting and nondepleting conditions in the conditional Ffh strain HDB51. Cells were fixed with formaldehyde and analyzed by fluorescence microscopy. mNeonGreen (NG) was used as a cytoplasmic control protein. Bars, 3 μm.

**FIG 5:**
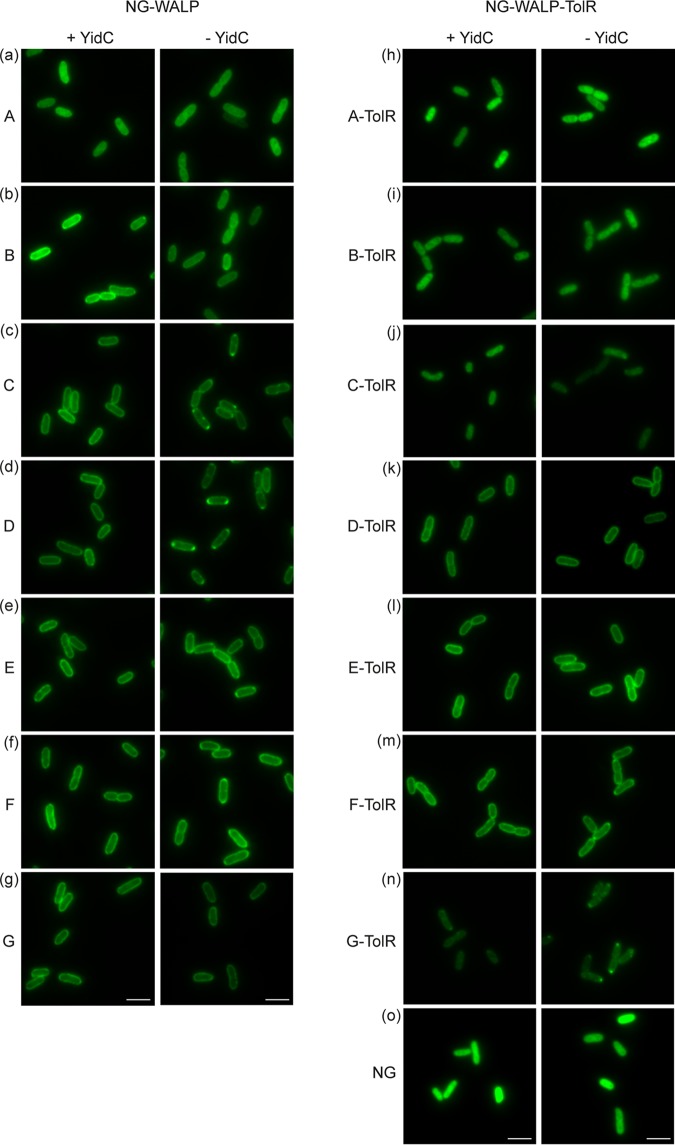
Role of YidC in the membrane insertion of NG-WALP and NG-WALP-TolR constructs. (a to o) NG-WALP and NG-WALP-TolR constructs were expressed under depleting and nondepleting conditions in the conditional YidC strain MK6s. Cells were fixed with formaldehyde and analyzed by fluorescence microscopy. mNeonGreen (NG) was used as a cytoplasmic control protein. Bars, 3 μm.

**FIG 6:**
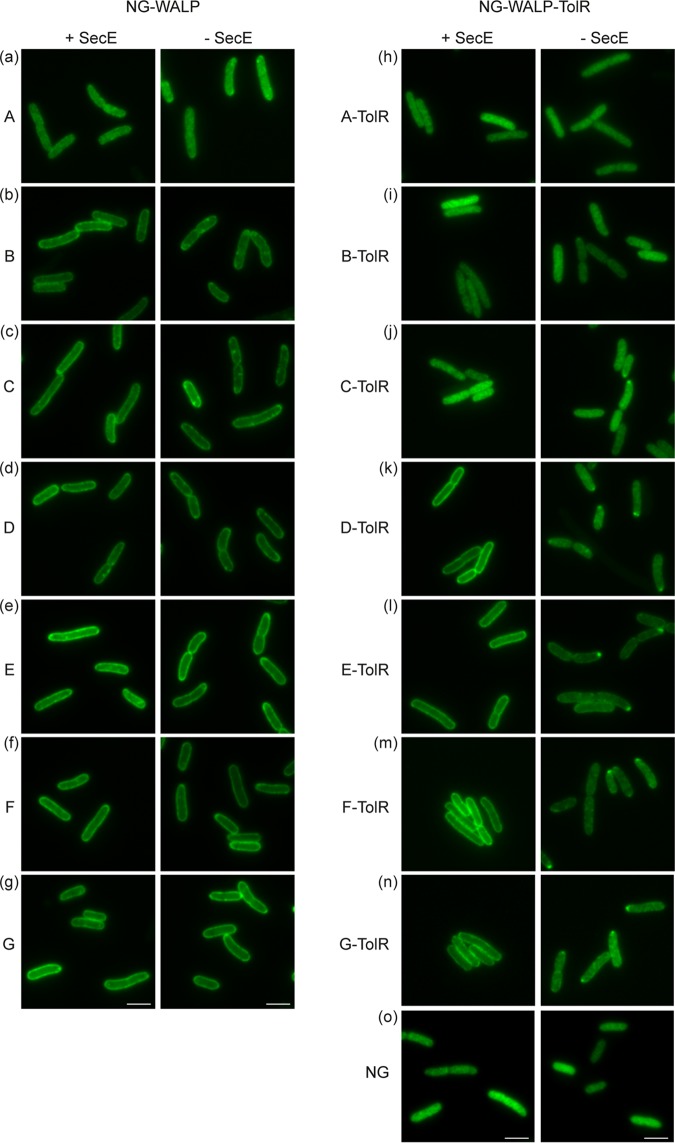
Role of SecYEG in the membrane insertion of NG-WALP and NG-WALP-TolR constructs. (a to o) NG-WALP and NG-WALP-TolR constructs were expressed under depleting and nondepleting conditions in a strain conditional for the essential SecYEG component SecE. Cells were fixed with formaldehyde and analyzed by fluorescence microscopy. mNeonGreen (NG) was used as a cytoplasmic control protein. Bars, 3 μm.

Strikingly, unlike the other NG-WALP constructs, NG-WALP-A did not localize at the membrane under nondepleted (plus l-arabinose) conditions (Fig. 4a), whereas it did in the wild-type strain MC4100 (Fig. 3a). Possibly, the low hydrophobicity of its TMD is at the threshold for anchoring in the membrane and its localization may depend on the specific genetic background of the strain used (see also below; Fig. 5a and Fig. 6a). Upon depletion of SRP, all other NG-WALP constructs were visible in aggregates that appeared membrane associated, whereas in addition halo formation indicative of membrane localization was observed for the WALP-C/D/E constructs of moderate hydrophobicity (Fig. 4c to e). Membrane localization of the NG-WALP-B construct appeared relatively inefficient in the presence of SRP and was further affected by the absence of SRP (Fig. 4b). Strikingly, the two constructs with the most hydrophobic TMDs, NG-WALP-F/G, did not display any halo formation upon SRP depletion and exclusively appeared as large spots within the cells (Fig. 4f and g). These results indicate that while part of the TAMP-TMDs of moderate hydrophobicity localize independently of SRP, the extremes of our set do strictly depend on SRP for targeting. Confirming this qualitative visual inspection, such a pattern is clearly apparent when the fluorescence profiles and center/border ratios of the fluorescence signal are compared (Fig. S4b to g and Fig. S7c).

10.1128/mBio.01580-19.4 FIG S4 Fluorescence profiles of HDB51 cells (a to n). Cross profiles of cells (*n*) were used to calculate the average fluorescence profile perpendicular to the cell axis of HDB51 cells expressing NG-WALP and NG-WALP-TolR constructs under nondepleted (+SRP) and depleted (-SRP) conditions. Cell count was determined automatically by the software, and variation stems from clumping of cells leading to different cell densities on the agarose pad. The filamentation of cells in the absence of Ffh additionally creates a big variation in cell count compared to cells that properly separate. Download FIG S4, TIF file, 2.7 MB. Copyright © 2019 Peschke et al. 2019 Peschke et al. This content is distributed under the terms of the Creative Commons Attribution 4.0 International license.

In the presence of SRP, the type II IMPs showed a fluorescence signal (Fig. 4h to n) similar to wild-type MC4100 (Fig. 3h to n) as expected. Under depleted conditions, membrane-associated spots appeared in all cells expressing type II IMP constructs, suggesting recruitment in aggregates (Fig. 4h to n). The distribution of the cytoplasmic control construct NG remained unaffected upon depletion of SRP (Fig. 4o), confirming that the results described above are independent of the reporter protein. The data indicate that the SRP plays a generic role in preventing the aggregation of all type II IMP constructs tested. Together with the fluorescence profile and the center/border ratio of the fluorescence signal (Fig. S4d to f and Fig. S7d), the data also imply that the constructs that are membrane localized under wild-type conditions (NG-WALP-TolR-D/E/F) require SRP for proper targeting.

### NG-WALPs insert independently of YidC irrespective of TMD hydrophobicity.

We previously observed that the NG-fused TMDs of E. coli TAMPs DjlC and Flk accumulate in polar aggregates in the absence of the membrane insertase YidC at the expense of halo formation, indicative of a moderate effect on membrane insertion (12). To investigate if the role of YidC is related to TMD hydrophobicity in TAMPs and how this compares to the corresponding type II IMPs, we expressed the two sets of constructs in the YidC depletion strain MK6s (20). The expression of YidC in this strain is controlled by an l-arabinose-inducible promoter. Depletion of YidC in the absence of l-arabinose was confirmed by Western blotting (Fig. S2c). Similarly to the strain HDB51, we confirmed that no overdepletion had occurred by monitoring the accumulation of pre-SurA, which is translocated and processed independently of YidC (Fig. S2c).

Similarly to strain HDB51 (Fig. 4a), NG-WALP-A showed cytoplasmic localization irrespective of the presence of YidC, indicating that the hydrophobicity of the TMD is below the threshold for membrane insertion in this genetic background (Fig. 5a). NG-WALP-B displayed halo formation at the cell envelope under depleted and nondepleted conditions (Fig. 5b), indicating that its membrane insertion is YidC independent. While the four constructs NG-WALP-C/D/E/F showed membrane localization similar to MC4100 in the presence of YidC (Fig. 2c to f and Fig. 5c to f), depletion of YidC induced the formation of polar spots, in particular for the moderately hydrophobic C and D variants. Importantly, halo formation was still apparent for all of the C to F polypeptides, indicating a facilitatory but not critical role for YidC in their membrane insertion. This conclusion is further supported by the fluorescence profile and the center/border ratio of the relevant fluorescent signals (Fig. S5c to f and Fig. S7e). Similarly to NG-WALP-B, no effect on membrane localization was observed for NG-WALP-G (Fig. 5g). Together, the results suggest that none of the TAMPs tested is strictly dependent on YidC, although YidC might improve the insertion efficiency of TAMPs with a moderately hydrophobic TMD (Fig. S7e).

10.1128/mBio.01580-19.5 FIG S5 Fluorescence profiles of MK6s cells (a to n)*. C*ross profiles of cells (*n*) were used to calculate the average fluorescence profile perpendicular to the cell axis of Mk6s cells expressing NG-WALP and NG-WALP-TolR constructs under nondepleted (+YidC) and depleted (-YidC) conditions. Cell count was determined automatically by the software, and variation stems from clumping of cells leading to different cell densities on the agarose pad. Download FIG S5, TIF file, 2.7 MB. Copyright © 2019 Peschke et al. 2019 Peschke et al. This content is distributed under the terms of the Creative Commons Attribution 4.0 International license.

For the set of type II IMPs, growth under nondepleting conditions gave localization patterns similar to MC4100. Overall, depletion of YidC had little effect on the distribution of the constructs tested, and we observed only some modest induction of polar spots for the polypeptides with the most hydrophobic TMDs, NG-WALP-TolR-F/G (Fig. 5h to n). None of the three membrane-localizing constructs NG-WALP-TolR-D/E/F appeared completely dependent on YidC for membrane insertion, irrespective of the difference in hydrophobicity of their TMDs (Fig. S5k to m and Fig. S7f). Confirming that these effects are independent of the reporter protein, NG showed cytoplasmic localization under depleted and nondepleted conditions (Fig. 5o).

### NG-WALPs do not require SecYEG for membrane insertion.

We previously found that compromising the SecYEG translocon had only minor effects on the membrane insertion of the NG-fused DjlC and Flk TMDs (12). To evaluate the role of the SecYEG translocon in the insertion of the TAMP and type II IMP series, we used a strain in which *secE* is under the control of an l-arabinose-inducible promoter (21). Depletion of SecE results in a rapid loss of the complete SecYE core of the translocon since SecY is degraded in the absence of SecE (22). To confirm successful inactivation of SecYEG, we monitored the accumulation of the precursor form of SurA that is known to require SecYEG for translocation (Fig. S2d).

Again, in this genetic background NG-WALP-A, containing the least hydrophobic TMD of the set, did not localize at the cell envelope even under nondepleting conditions (Fig. 6a) as described above for the conditional Ffh and YidC strains. The other NG-WALP constructs showed clear halo formation independent of the SecYEG translocon with occasional weak polar or circumferential spots under depletion conditions (Fig. 6b to g). Quantification of the data corroborates the Sec-independent membrane localization of all model TAMPs, irrespective of TMD hydrophobicity (Fig. S6b to g and Fig. S7g). Of note, we found that only 1 h of SecYEG depletion caused a severe reduction of YidC levels in the cells (Fig. S2d), suggesting that the observed minor effects might be secondary and due to YidC depletion as discussed before (19).

10.1128/mBio.01580-19.6 FIG S6 Fluorescence profiles of CM124 cells (a to n). Cross profiles of cells (*n*) were used to calculate the average fluorescence profile perpendicular to the cell axis of CM124 cells expressing NG-WALP and NG-WALP-TolR constructs under nondepleted (+SecE) and depleted (-SecE) conditions. Cell count was determined automatically by the software, and variation stems from clumping of cells leading to different cell densities on the agarose pad. Download FIG S6, TIF file, 2.7 MB. Copyright © 2019 Peschke et al. 2019 Peschke et al. This content is distributed under the terms of the Creative Commons Attribution 4.0 International license.

In marked contrast, the type II IMPs NG-WALP-TolR-D/E/F that are membrane localized in the presence of SecYEG (Fig. 6k to m), as also seen with wild-type MC4100 cells (Fig. 2d to f), showed severe defects in halo formation upon depletion. This trend is further supported by analysis of the fluorescence profile and center/border ratio of the fluorescence signals (Fig. S6k to m and Fig. S7h), and we conclude that the membrane insertion of these proteins is impaired. Surprisingly, NG-WALP-TolR-G, which showed no halo formation in nondepleted Ffh and YidC conditional strains (Fig. 4n and Fig. 5n), displayed clear halo formation under nondepleted conditions in CM124 (Fig. 6n, Fig. S6n, and Fig. S7h), again suggesting that the TMD hydrophobicity thresholds for membrane insertion may be somewhat strain dependent. Upon SecYEG depletion, NG-WALP-TolR-G did not localize with membranes, and spots appeared at the cell poles (Fig. 6n), which was similar to the other membrane-associated polypeptides of this series.

Since we found no effect of YidC depletion on the localization of the NG-WALP-TolR constructs (Fig. 5h to n), we reasoned that the effects observed upon SecE depletion are most likely caused by a compromised SecYEG translocon rather than by secondary depletion of YidC (Fig. S2d). As seen in the wild-type MC4100 strain (Fig. 3b), NG-WALP-TolR-A/B/C appear incapable of membrane insertion and showed a cytoplasmic distribution in the presence and absence of SecYEG (Fig. 6h to j). Combined, the results show a clear dependency on the SecYEG translocon for proper membrane insertion of NG-WALP-TolR-D/E/F/G that seems independent of the overall hydrophobicity of the TMD. The soluble protein NG remained unaffected in the presence or absence of SecYEG, indicating that the observed effects are dependent on the TMD and C-terminal tail (Fig. 6o).

### TAMPs with TMDs of moderate hydrophobicity are capable of unassisted insertion *in vitro*.

**FIG 7:**
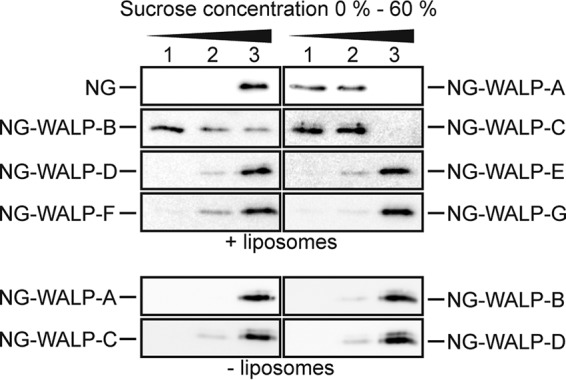
NG-WALPs with low-hydrophobicity TMDs can insert spontaneously into protein-free liposomes. Purified NG and NG-WALPs were incubated with protein-free liposomes consisting of 75% PE and 25% PG. Subsequently, samples were subjected to sucrose density gradient centrifugation, and three fractions were taken from top to bottom (1 to 3). Fractions were analyzed by Western blotting using anti-His serum.

Studies on the biogenesis of eukaryotic TAMPs revealed that TAMPs containing moderately hydrophobic TMDs are capable of spontaneous insertion into protein-free liposomes (6, 23). We found that only NG-WALP-C was strictly dependent on DnaJ/K (Fig. 3c) and only NG-WALP-F/G were strictly dependent on SRP (Fig. 4f and g), whereas none of the NG-WALPs strictly required either YidC (Fig. 5a to g) or SecYEG (Fig. 6a to g) for efficient membrane insertion. These results leave open the possibility that at least some NG-WALPs are capable of spontaneous membrane insertion, as previously reported for a small subset of eukaryotic TAMPs (6). To investigate this possibility, we performed liposome flotation assays. Purified recombinant NG-WALPs were mixed with protein-free liposomes consisting of 75% phosphatidylethanolamine (PE) and 25% phosphatidylglycerol (PG), to approximate the lipid composition of the E. coli inner membrane (24). Subjecting these samples to sucrose gradient centrifugation allows separation of floating liposomes and associated proteins from soluble, non-membrane-associated proteins that do not float. Soluble NG served as a negative control, and it remained in the bottom fraction of the density gradient (Fig. 7). NG-WALP-A/B/C floated with liposomes, as indicated by their presence in the top and middle fractions, albeit some NG-WALP-B was also recovered from the bottom fraction (Fig. 7). The remaining NG-WALP polypeptides with higher TMD hydrophobicity all remained in the bottom fraction of the density gradients (Fig. 7). The results suggest that NG-WALPs with moderately hydrophobic TMDs are capable of unassisted association with and/or insertion into protein-free liposomes, similarly to the eukaryotic TAMPs cytochrome *b*_5_ and PTB1B (23).

## DISCUSSION

The biogenesis of TAMPs is complicated by the fact that their TMD cannot engage chaperones or membrane insertases cotranslationally, thus increasing the risk of misfolding and aggregation upon release of the full-length protein from the ribosome. In eukaryotes, TAMPs follow discrete but partly redundant targeting and insertion pathways to address this challenge, as exemplified by the GET/TRC40 pathway for TAMP biogenesis (5, 9, 25). Interestingly, mammalian SRP was found to promote the membrane targeting of TAMPs with a particularly hydrophobic TMD via an unusual posttranslational mechanism (17). In prokaryotes, much less is known about the routing of TAMPs.

Here, we have analyzed the impact of the hydrophobicity of the TMD on the localization of model TAMPs in E. coli. We have primarily used fluorescence microscopy, because it appears to inform more reliably on localization than classical fractionation due to the tendency of aggregated mislocalized membrane proteins to sediment with membranes during centrifugation. For this, we constructed synthetic TAMPs with a cytosolic N-terminal fluorescent reporter fused to a synthetic WALP TMD peptide of varying hydrophobicity (A to G in order of increasing hydrophobicity) to mediate membrane targeting and anchoring. The same constructs were also extended with the C-terminal periplasmic domain of the TolR protein to convert them into regular type II IMPs and serve as a control for position-specific effects of the TMD. All constructs were tested in strains conditional for the expression of known targeting and insertion factors, as previous site-directed cross-linking had not revealed any novel interacting partners of the TMD in the model TAMPs DjlC and Flk (12).

Strikingly, we now show that the hydrophobicity and the relative position of a single TMD have a huge impact on both the membrane localization of the protein and the cellular machinery that it requires to reach this location. We observed that TMDs of moderate hydrophobicity are perfectly capable of inserting a TMD in the correct N-in C-out orientation in the inner membrane when positioned in the context of the TAMP (at the C terminus). In contrast, the type II IMP constructs in which the same TMDs were placed internally remained cytosolic. Consistent with previous studies on TMDs with an N-in, C-out orientation in “regular” IMPs (26), the latter constructs required a strongly hydrophobic signal anchor for efficient membrane insertion and the translocation of the extended C-terminal domain. We found that the membrane insertion-competent members of these extended type II IMPs require SRP for targeting and the Sec translocon for membrane insertion and translocation, similarly to the endogenous E. coli type II IMP FtsQ (27, 28).

The membrane insertase YidC has been shown to be strictly required for the Sec-independent membrane insertion of F_0_C (subunit c of the F_1_F_0_-ATPase) and the phage coat proteins M13 and Pf3 (29–31). These proteins are rather small, and like TAMPs, they do not contain large periplasmic domains. However, all NG-WALP constructs analyzed showed membrane localization upon depletion of YidC. In the case of NG-WALP-B/C/D/E/F, we did find some evidence for protein aggregates at the cell poles, arguing that YidC may improve the efficiency of TAMP insertion without being absolutely required. Our data do not exclude the possibility that YidC is the preferred insertase for these constructs, a role that can be compensated for by other, yet-unidentified insertion factors when YidC is absent. In agreement with a physiological, albeit not critical, role for the YidC insertase, we previously demonstrated an interaction between YidC and the TMDs of the natural TAMPs DjlC and Flk by site-specific photo-cross-linking (12). In the case of our model type II IMPs, we did not observe a requirement for YidC, with the exception of minor aggregate formation at the cell poles for NG-WALP-TolR-F/G. Corroborating our data, proteomics using YidC-depleted E. coli have shown that IMPs with sizeable periplasmic domains are unaffected by the depletion of YidC (32).

Likewise, none of the seven NG-WALP TAMPs required a functioning SecYEG translocon for membrane insertion. This is consistent with the reported Sec-independent insertion of the natural TAMPs DjlC, Flk, and SciP (11, 33). Analysis of the inner membrane proteome of cells depleted of the essential SecYEG component SecE has revealed that while the level of a considerable number of IMPs was reduced, an equal number of IMPs remained unaffected or were increased (34). Most of the unaffected or upregulated IMPs contained only one or two TMDs and/or lacked large periplasmic domains, consistent with the Sec-independent insertion of TAMPs that we observe.

The assessment of SRP dependence was complicated by the aggregates that accumulate in the cytoplasm. All WALPs and corresponding type II IMPs showed fluorescent dots upon expression in the absence of SRP. We believe that the endogenous cytosolic protein aggregates, which are known to form in the absence of SRP and do contain cytosolic as well as secreted and membrane proteins (35), may recruit some fraction of the newly synthesized TAMPs and thereby generate the fluorescent species that we observe. In other words, kinetic partitioning of newly synthesized TAMP precursors may occur between membrane localization, where this is possible in the absence of SRP-mediated protein targeting, and binding to preexisting cytosolic aggregates. Such behavior could also complicate the interpretation of classical fractionation studies and lead to an overestimation of the impact of SRP depletion. In this study, we find that not all TMDs imposed strict SRP dependence on TAMP targeting, but the most hydrophobic do, consistent with the SRP dependency of the authentic TAMP Flk, which also carries an extremely hydrophobic TMD (12). The effect of SRP depletion on NG-WALP-B with a TMD of low hydrophobicity is difficult to explain and may relate to specific structural features of this sequence that increase recruitment to aggregates formed under these conditions. In contrast, all corresponding type II IMPs were dependent on SRP for membrane targeting, as might be expected.

Possibly, C-terminal TMDs can more easily engage alternative chaperones such as DnaJ/K. For instance, the NG-WALP-C construct was primarily localized in aggregates in the absence of DnaJ/K. Surprisingly, this construct was perfectly able to associate with liposomes without any assistance of proteinaceous factors similar to NG-WALP-A and -B. *In vivo*, DnaJ/K might help to prevent aggregation of this construct in the crowded cytosol, extending the time window in which it can insert unassisted or recruit alternative targeting factors or membrane insertases. This behavior is reminiscent of the mammalian TAMP Cytb5, which also carries a moderately hydrophobic TMD and is capable of unassisted insertion in liposomes but associates with Hsc70 in lysates (6, 36). Mammalian TAMPs with a more hydrophobic TMD did not insert in liposomes, which was attributed to their stronger tendency to aggregate in the absence of chaperones. Following a similar reasoning, NG-WALP-D/E/F/G might be too hydrophobic to remain translocation competent in the *in vitro* liposome assay. While F/G were shown to require SRP *in vivo*, the relative independence of D/E of both DnaJ/K and SRP might imply involvement of an alternative chaperone for these constructs or an intrinsic solubility in the *in vivo* physiological context. The type II IMPs localized correctly *in vivo* in the absence of DnaJ/K, although occasional spots may again hint at attraction to aggregates, a feature that appears independent of TMD hydrophobicity in this context.

In conclusion, in E. coli, as in eukaryotes, TAMPs seem to have different constraints for membrane targeting and insertion than regular membrane proteins with more TMDs and/or large periplasmic loops that have to be translocated across the inner membrane. Given that even moderately hydrophobic TMDs (GRAVY, 1.04 to 1.57) can drive TAMP insertion, it will be worthwhile revisiting current bioinformatic estimates of the number of TAMPs that exist in E. coli and other prokaryotes (10). Of note, the algorithms for TAMP identification use a threshold for TMD hydrophobicity that is based on regular IMPs, which we had found to need a more hydrophobic TMD for SRP-mediated targeting to the Sec translocon. In contrast, as for eukaryotes, TAMP biogenesis in prokaryotes may follow multiple alternative pathways that provide functional redundancy (cf reference 25). Furthermore, the membrane localization of TAMPs with a TMD of low hydrophobicity may occur via an unassisted mechanism, as evidenced by efficient *in vitro* association with liposomes. Conceivably, this pathway makes sense for prokaryotes since TAMPs can enter only the cytoplasmic membrane. In eukaryotes, however, mistargeting of a TAMP to another organelle membrane is an issue that may require more elaborate parallel sorting mechanisms.

In summary, our study highlights position-specific effects of TMD function in E. coli IMPs. Hence, a TMD of moderate hydrophobicity may function in targeting and membrane insertion when located at the C terminus, whereas the same TMD located at an internal position may not mediate membrane insertion. Likewise, it would appear that the basis for precursor discrimination between different targeting/insertion pathways is also position dependent. Thus, a TMD that meets the hydrophobicity requirements for functioning both at the C terminus of an IMP and internally in a type II IMP may use different mechanisms to achieve efficient membrane insertion. Interestingly, a recent study describes a subset of effector proteins that are secreted via the type III and type IV secretion systems and yet have predicted TMDs of moderate hydrophobicity (37). It was shown that the predicted TMDs can indeed insert in the inner membrane when they are grafted into a polytopic IMP, suggesting a similar context dependency of TMD functioning.

## MATERIALS AND METHODS

### Strains and growth conditions.

E. coli TOP10F′ was used as a cloning strain, and E. coli BL21(DE3) was used for protein purification. The Ffh depletion strain HDB51 (18) and the YidC depletion strain MK6s (20) were grown overnight in sodium phosphate-buffered LB supplemented with 0.4% d-glucose and 0.2% l-arabinose. Subsequently, cultures were back-diluted to an optical density at 660 nm (OD_660_) of 0.01 and grown for 3 h in the presence of 0.4% d-glucose and 0.2% l-arabinose for nondepleting or with 0.4% d-glucose for depleting conditions before protein expression was induced with 0.5 mM isopropyl-β-d-1-thiogalactopyranoside (IPTG) for 1 h. The SecE depletion strain CM124 (21) was grown overnight in sodium phosphate-buffered LB supplemented with 0.2% glycerol and 0.4% l-arabinose. The cultures were back-diluted to an OD_660_ of 0.1 and grown for 1 h in the presence of 0.2% glycerol and 0.4% l-arabinose for nondepleting conditions and with 0.2% glycerol and 0.4% d-glucose for depleting conditions, and subsequently protein expression was induced as described above. The DnaJ/K-knockout strain MC4100*ΔdnaJK* (16) and its isogenic wild-type MC4100 were grown in LB overnight at 30°C, subsequently back-diluted to an OD_660_ of 0.05, and grown until reaching an OD_660_ of 0.3 before protein expression was induced as above.

### Plasmid construction.

The plasmids used in this study and the oligonucleotides used to create them are listed in Tables S1 and S2 in the supplemental material. His-NG-WALP-A to -G were constructed by adding the DNA sequence of the WALP TMDs to His-NG in two PCR steps and cloned into the spectinomycin-resistant expression vector pSE(p15a)spR and into the kanamycin-resistant expression vector pSE(p15a)kmR via NcoI and BamHI. To construct the pET16b His-NG-WALP derivatives, the respective NG-WALP sequences were cut from the pSE(p15a) vector via NdeI and BamHI and inserted into pET16b. The His-NG-WALP-TolR constructs were created by overlap extension PCR. Sequences encoding His-NG-WALPs and the periplasmic domain of TolR were amplified with overlapping flanking regions that were used as the template DNA to create the fusion constructs. The resulting His-NG-WALP-TolR constructs were inserted into the expression vectors pSE(p15a)spR and pSE(p15a)kmR via NcoI and BamHI. To provide His-NG-WALP-B/C/G with a C-terminal opsin tag, the respective constructs were used as a template for PCRs with a reverse primer containing the sequence of the opsin tag.

10.1128/mBio.01580-19.8 TABLE S1 List of PCR primers used to create the constructs in this study. Download Table S1, DOCX file, 0.01 MB. Copyright © 2019 Peschke et al. 2019 Peschke et al. This content is distributed under the terms of the Creative Commons Attribution 4.0 International license.

### Cell fractionation and membrane extraction.

E. coli MC4100 harboring derivatives of pSE(p15a) was grown to an OD_660_ of 0.3 at 37°C, and protein expression was induced for 1 h with 0.5 mM isopropyl-β-d-1-thiogalactopyranoside (IPTG). After induction, cells were harvested for 10 min at 4,600 × *g* at 4°C and lysed at 1.82 × 10^8^ Pa using the OneShot cell disruptor (Constant Systems Ltd.). Cell debris and unlysed cells were spun down for 10 min at 4,600 × *g* at 4°C, and crude membranes were subsequently isolated from the supernatant for 1 h at 347,000 × *g* at 4°C. The membranes were resuspended in PBS, split into two fractions, mixed with either PBS or 2% *N*-dodecyl-β-d-maltoside (DDM), and incubated on a turning wheel for 1 h at 4°C. Subsequently, the samples were spun for 45 min at 190,000 × *g* at 4°C. Supernatant fractions were trichloroacetic acid (TCA) precipitated and resuspended in SDS sample buffer, and pellet fractions were directly dissolved in SDS sample buffer.

### Proteinase K accessibility in spheroplasts.

E. coli MC4100 cells were grown to an OD_660_ of 0.3, and subsequently protein expression of N-terminally His-tagged and C-terminally opsin-tagged NG-WALPs and N-terminally His-tagged NG-WALP-TolRs was induced with 0.5 mM IPTG for 30 min. Cells were harvested in 1.5-ml microcentrifuge tubes for 2 min at 16,000 × *g*. Cell pellets were resuspended in 100 mM Tris (pH 8.0) and 1 M sucrose. After addition of 1 mM EDTA (pH 8.0) and 5 μg/ml lysozyme, cells were incubated at room temperature (RT) for 15 min. Spheroplast formation was stopped by addition of MgCl_2_ to a final concentration of 20 mM. After harvesting for 2 min at 6,000 × *g* at 4°C, the stabilized spheroplasts were resuspended in 100 nM Tris (pH 8.0), 250 mM sucrose, and 20 mM MgCl_2_. ProtK (or buffer for mock treatment) was added to a final concentration of 2.5 μg/ml with or without Triton X-100 to a final concentration of 1% and incubated for 20 min at 25°C. ProtK was inactivated for 10 min on ice with 100 mM phenylmethylsulfonyl fluoride (PMSF).

### Fluorescence microscopy.

Cells were fixed with 2.8% formaldehyde for 10 min on ice, washed twice with PBS, and stored at 4°C. For analysis, 0.03 OD units of cells was immobilized on 1% (wt/vol in PBS) Noble agar pads and imaged using an Olympus BX-60 fluorescence microscope equipped with a 100×/numerical-aperture (NA) 1.35 oil objective and a CoolSNAP *fx* (Photometrics) charge-coupled device (CCD) camera. The software ImageJ-MicroManager was used for image acquisition. Analysis of microscopic images was performed with ImageJ, plugin ObjectJ, Coli-Inspector (38), and CrossProfilesMacro1.0. ImageJ in conjunction with the plugins ObjectJ and Coli-Inspector was used for automatic measurements of cell count, cell length, and cell diameter. The macro CrossProfilesMacro1.0 qualitatively analyzes the ratio between membrane and cytoplasmic localization of fluorescent signals by creating intensity profiles perpendicular to the cell axes for cells that have been analyzed by Coli-Inspector.

### Protein purification.

E. coli BL21(DE3) harboring derivatives of pET16b was grown to an OD_660_ of 0.3 at 37°C, and protein expression was induced overnight at 24°C with 0.4 mM IPTG. The cells were harvested for 10 min at 4,600 × *g* at 4°C, resuspended in buffer A (50 mM Na_2_HPO_4_, 300 mM NaCl, 10% glycerol), and lysed using the OneShot cell disruptor (Constant Systems Ltd.) at 1.82 × 10^8^ Pa. Cell debris and unlysed cells were spun down for 10 min at 4,600 × *g* at 4°C, and crude membranes were spun down for 1 h at 293,100 × *g* at 4°C. The membranes were resuspended in buffer A, DDM was added to 2%, and membranes were solubilized overnight at 4°C on a turning wheel. Unsolubilized material was spun down for 45 min at 300,000 × *g* at 4°C. The supernatant was diluted to 1% DDM and incubated with Talon Superflow (GE Healthcare) for 2 h at 4°C on a turning wheel. After removal of the flowthrough by centrifugation using Pierce Spin columns (Thermo Fisher Scientific), the resin was washed with 30 bed volumes of buffer W (50 mM Na_2_HPO_4_, 300 mM NaCl, 10% glycerol, 0.1% DDM, 20 mM imidazole). His-tagged NG-WALP chimeras were eluted from the resin using buffer E (50 mM Na_2_HPO_4_, 300 mM NaCl, 10% glycerol, 0.1% DDM, 400 mM imidazole). To remove the imidazole, the samples were applied to PD SpinTrap G-25 desalting columns (GE Healthcare).

### Liposome insertion assay.

l-α-Phosphatidylethanolamine (PE) and l-α-phosphatidylglycerol (PG) were purchased in powder form from Avanti Polar Lipids. PE and PG were mixed (mol/mol, 75%/25%) in a round-bottom Erlenmeyer flask and dissolved in chloroform. A dry lipid film was created under vacuum in a 40°C water bath using a rotor evaporator. The resulting lipid film was resuspended in PBS by vortexing with glass beads (5-mm diameter; Sigma-Aldrich). The lipid suspension was then passed 15 times through a 0.2-μm polycarbonate membrane and subsequently 15 times through a 0.05-μm polycarbonate membrane using a Mini-Extruder (all from Avanti Polar Lipids). The homogeneity of the size of the liposomes was confirmed by dynamic light scattering (DLS).

Fifteen microliters of PE/PG liposomes (12 mg/ml) was mixed with 200 ng of purified His-NG-WALP chimeras, adjusted to a total volume of 20 μl with PBS, and incubated for 90 min at 37°C. The reaction mixture was then mixed with 50 μl of 60% (wt/vol) sucrose in PBS and transferred to a 200-μl ultracentrifuge tube. The sample was then carefully overlayered with 60 μl of 30% (wt/vol) sucrose in PBS and 60 μl PBS, respectively. The samples were spun for 30 min at 250,000 × *g* at 4°C. Three fractions of 63 μl each were taken from the top of the gradient to the bottom, mixed with SDS sample buffer, and analyzed by Western blotting.

10.1128/mBio.01580-19.9 TABLE S2 List of plasmid constructs used in this study. Download Table S2, DOCX file, 0.01 MB. Copyright © 2019 Peschke et al. 2019 Peschke et al. This content is distributed under the terms of the Creative Commons Attribution 4.0 International license.
