# Supplementary figures and images for: Distinct Requirements for Tail-Anchored Membrane Protein Biogenesis in Escherichia coli
Source: mBio. 2019 Oct 15;10(5):e01580-19. doi: 10.1128/mBio.01580-19 (PMC6794478; doi:10.1128/mBio.01580-19)

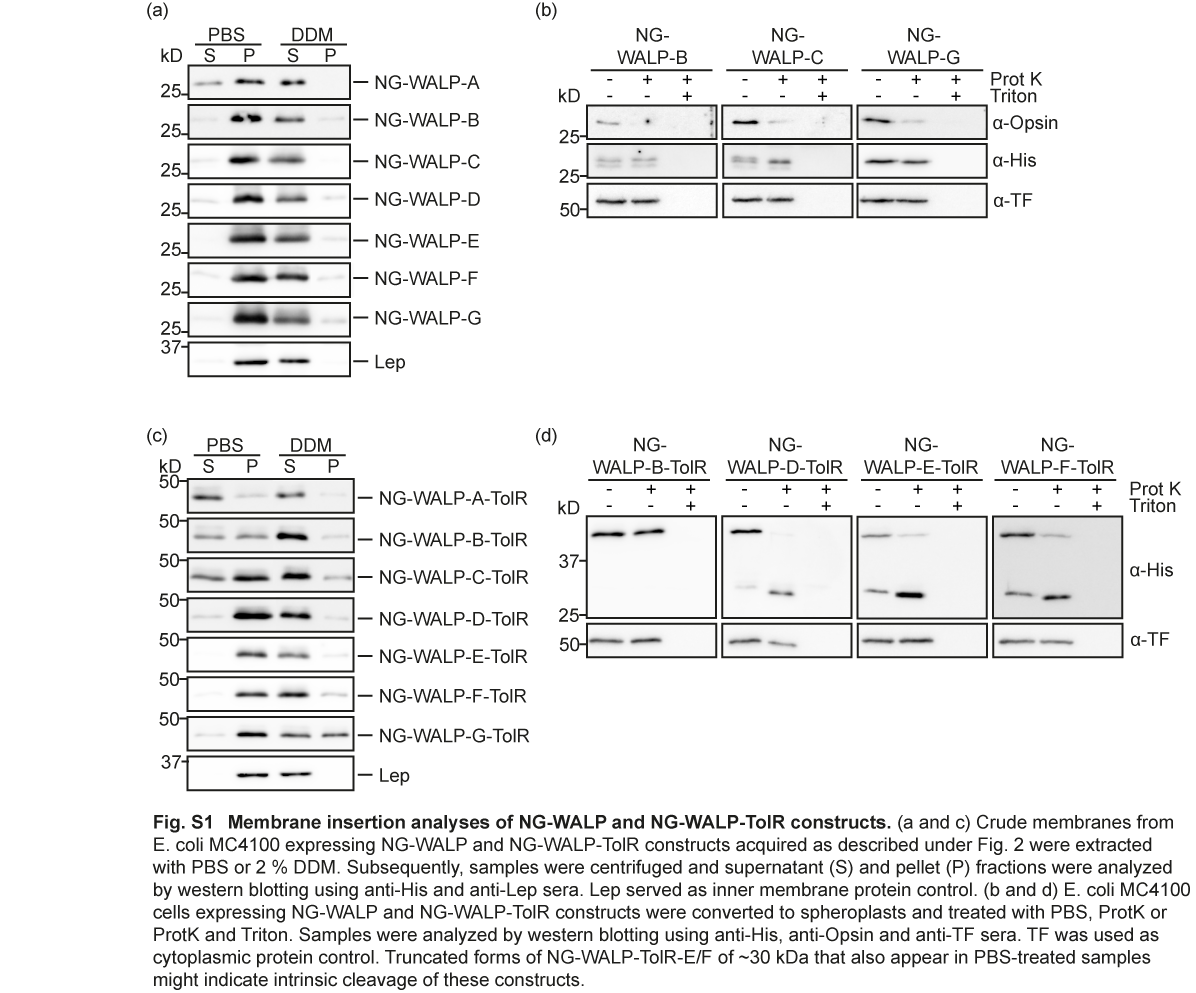

Supplement: FIG S1 [file mBio.01580-19-sf001.tif]

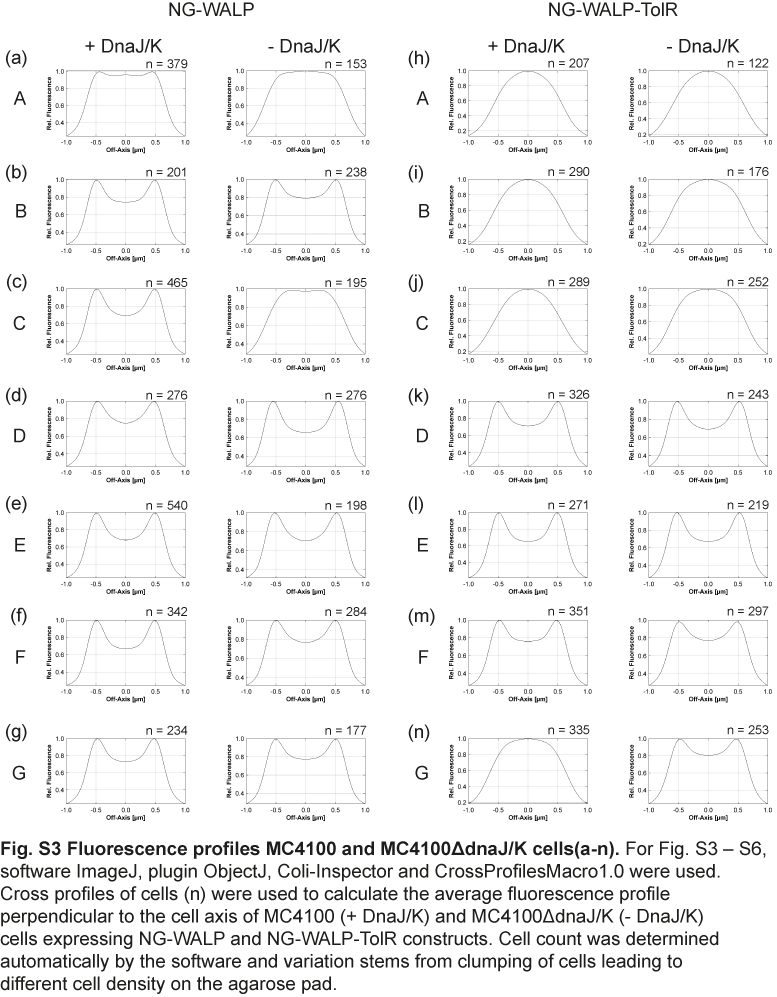

Supplement: FIG S3 [file mBio.01580-19-sf003.tif]

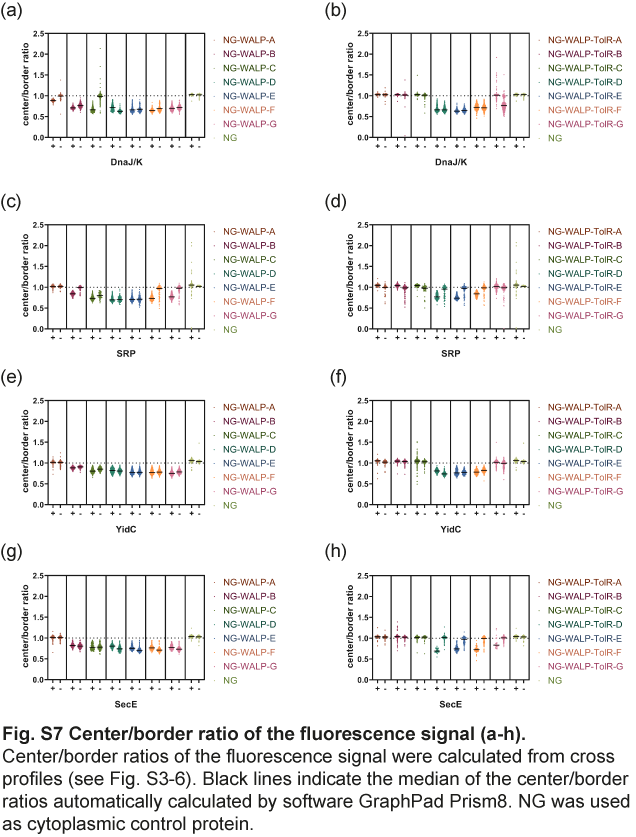

Supplement: FIG S7 [file mBio.01580-19-sf007.tif]

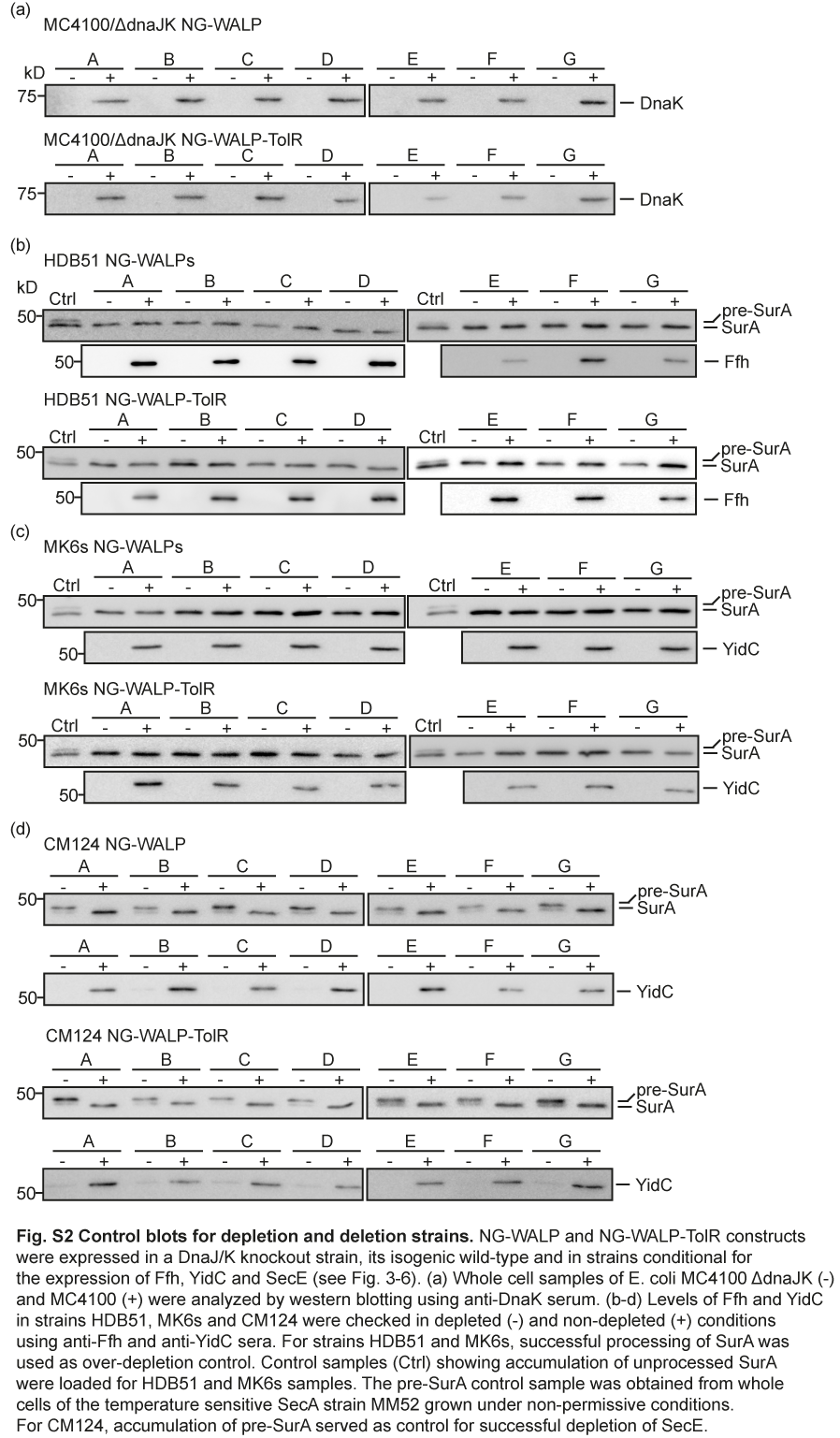

Supplement: FIG S2 [file mBio.01580-19-sf002.tif]

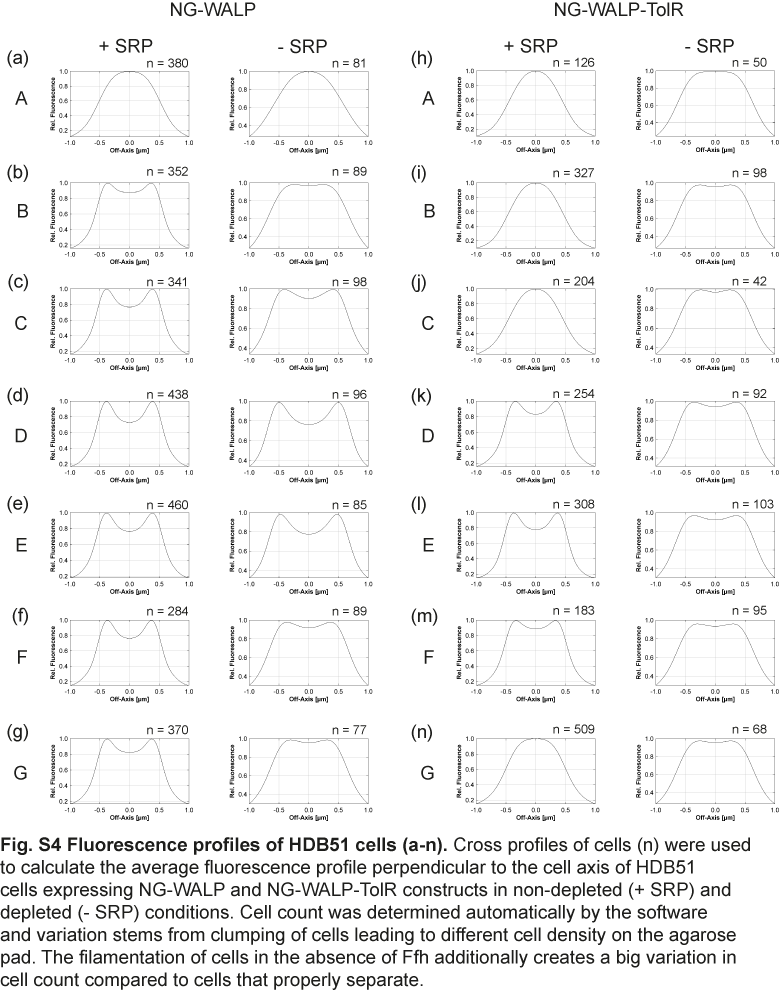

Supplement: FIG S4 [file mBio.01580-19-sf004.tif]

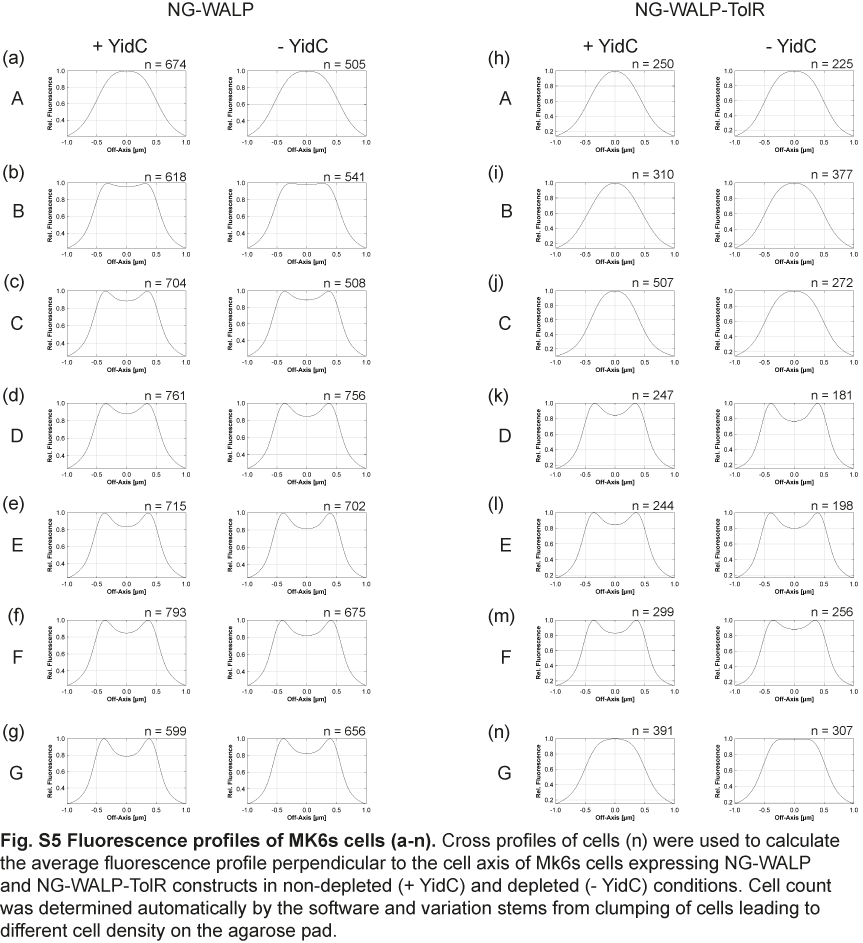

Supplement: FIG S5 [file mBio.01580-19-sf005.tif]

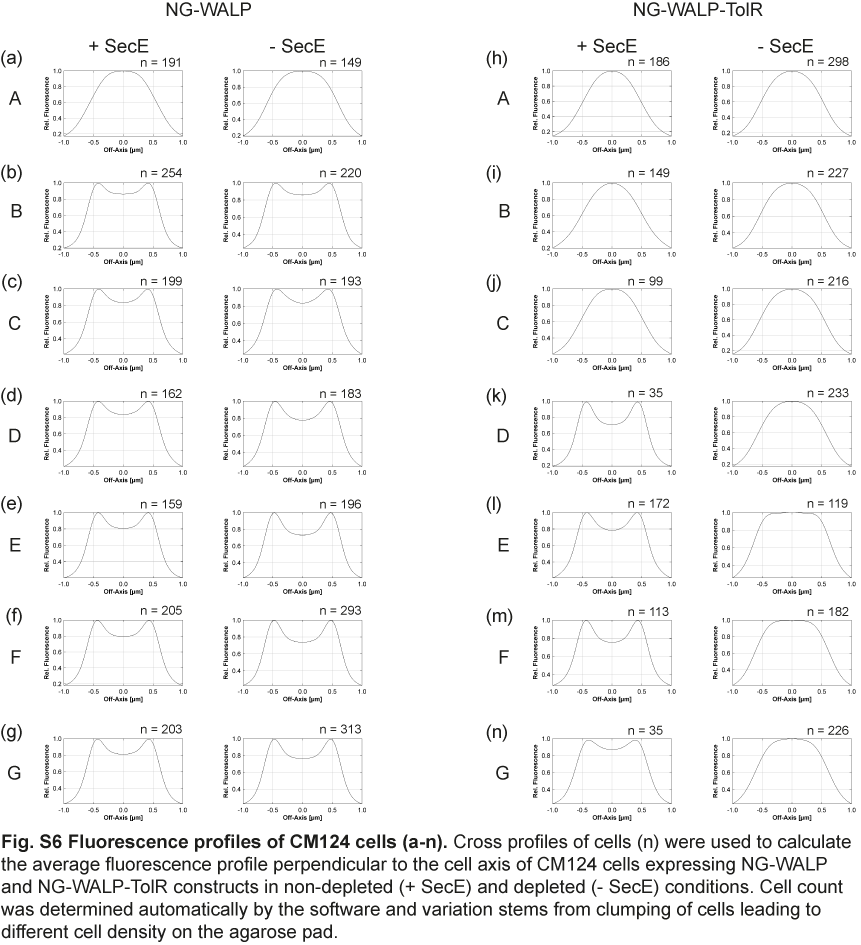

Supplement: FIG S6 [file mBio.01580-19-sf006.tif]
