# Supplementary material for: Distinct Requirements for Tail-Anchored Membrane Protein Biogenesis in Escherichia coli
Source: mBio. 2019 Oct 15;10(5):e01580-19. doi: 10.1128/mBio.01580-19 (PMC6794478; doi:10.1128/mBio.01580-19)
Supplement: TABLE S1 [file mBio.01580-19-st001.docx]

| **Name** | **Purpose** | **Sequence (5' → 3')** |
| --- | --- | --- |
| His-NG NcoINdeI fw | forward primer for His-NG-WALP constructs | CAACCATGGGACACCACCACCACCACCATATGGTGAGCAAGGGCGAG |
| WALP A rv1 | reverse primer 1 for NG-WALP A | GCTGCGGAAGCAGCCGCCGCTGCCCACCATGACTTTTTAGAACCGCCGCCTCCGAATTCCTTGTA |
| WALP A BamHI rv | reverse primer 2 for NG-WALP A | CAATTGGATCCTTATGATCCCCACCAGGCAGCTGCTGCAGCAGAGGCCGCAGCAGCTGCGGAAGCA |
| WALP B rv1 | reverse primer 1 for NG-WALP B | GCTGCTGCAGCAGCCGCCGCTGCCCACCATGACTTTTTAGAACCGCCGCCTCCGAATTCCTTGTA |
| WALP B BamHI rv | reverse primer 2 for NG-WALP B | CAATTGGATCCTTATGATCCCCACCAGGCAGCTGCTGCAGCGGCGGCCGCGGAAGCTGCTGCAGCA |
| WALP C rv1 | reverse primer 1 for NG-WALP C | GCTGCAGCAGCCGCCGCTGCCCACCATGACTTTTTAGAACCGCCGCCTCCGAATTCCTTGTA |
| WALP C BamHI rv | reverse primer 2 for NG-WALP C | CAATTGGATCCTTATGATCCCCACCAGGCAGCTGCTGCAGCGGCGGCCGCAGCAGCTGCTGCAGCAGCC |
| WALP D rv1 | reverse primer 1 for NG-WALP D | GCTGCTGCTAGAGCCGCCGCTGCCCACCATGACTTTTTAGAACCGCCGCCTCCGAATTCCTTGTA |
| WALP D BamHI rv | reverse primer 2 for NG-WALP D | CAATTGGATCCTTATGATCCCCACCAGGCAGCTGCTGCTAAGGCGGCCGCAAGAGCTGCTGCTAGA |
| WALP E rv1 | reverse primer 1 for NG-WALP E | GCCAGAGCGAGCGCTAGGGCGGCCCACCATGACTTTTTAGAACCGCCGCCTCCGAATTCCTTGTA |
| WALP E BamHI rv | reverse primer 2 for NG-WALP E | CAATTGGATCCTTATGATCCCCACCAAGCAGCCAGCGCCAAGGCTAAGGCAAGAGCCAGAGCGAGC |
| WALP F rv1 | reverse primer 1 for NG-WALP F | CGCCAAGGCTAATGCGAGTAGTAGCCACCATGACTTTTTAGAACCGCCGCCTCCGAATTCCTTGTA |
| WALP F BamHI rv | reverse primer 2 for NG-WALP F | CAATTGGATCCTTATGATCCCCACCACAATAATAACGCTAGTGCCAGAGCAAGCGCCAAGGCTAA |
| WALP G rv1 | reverse primer 1 for NG-WALP G | AATAACAGCAGGAGTAGTAGCCACCATGACTTTTTAGAACCGCCGCCTCCGAATTCCTTGTA |
| WALP G BamHI rv | reverse primer 2 for NG-WALP G | CAATTGGATCCTTATGATCCCCACCACAACAAAAGTAACAATAGGAGAAGCAGCAGCAATAACAGCAGG |
| WALP A-D TolR rv | reverse primer for NG-WALP A-D for TolR fusion | AGTAGCGTCTGGCAGATCGACCTCCACGCTCTGCCACCAGGCAGC |
| WALP E TolR rv | reverse primer for NG-WALP E for TolR fusion | AGTAGCGTCTGGCAGATCGACCTCCACGCTCTGCCACCAAGCAGC |
| WALP F TolR rv | reverse primer for NG-WALP F for TolR fusion | AGTAGCGTCTGGCAGATCGACCTCCACGCTCTGCCACCACAATAA |
| WALP G TolR rv | reverse primer for NG-WALP G for TolR fusion | AGTAGCGTCTGGCAGATCGACCTCCACGCTCTGCCACCACAACAA |
| ppTolR fw | forward primer for TolR periplasmic domain | GATCTGCCAGACGCTACT |
| TolR BamHI rv | reverse primer for TolR periplasmic domain | CAATTGGATCCTTAGATAGGCTGCGT |
| WALP-opsin BamHI rv | reverse primer to add opsin-tag to WALP B/C/G | CAATTGGATCCTTAGCCCGTCTTGTTGGAGAAAGGCACGTAGAAGTTTGGGCCT |

**TABLE S1** List of PCR primers used to create the constructs in this study.
