# Supplementary material for: Distinct Requirements for Tail-Anchored Membrane Protein Biogenesis in Escherichia coli
Source: mBio. 2019 Oct 15;10(5):e01580-19. doi: 10.1128/mBio.01580-19 (PMC6794478; doi:10.1128/mBio.01580-19)
Supplement: TABLE S2 [file mBio.01580-19-st002.docx]

**TABLE S2** List of plasmid constructs used in this study.

| **Name** | **Resistance** | **Source** |
| --- | --- | --- |
| pSE(p15a) His-NG spR | Spectinomycine | Peschke et. al. 2018 |
| pSE(p15a) His-NG kmR | Kanamycin | Peschke et. al. 2018 |
| pSE(p15a) His-NG-WALP-A spR | Spectinomycine | This study |
| pSE(p15a) His-NG-WALP-B spR | Spectinomycine | This study |
| pSE(p15a) His-NG-WALP-C spR | Spectinomycine | This study |
| pSE(p15a) His-NG-WALP-D spR | Spectinomycine | This study |
| pSE(p15a) His-NG-WALP-E spR | Spectinomycine | This study |
| pSE(p15a) His-NG-WALP-F spR | Spectinomycine | This study |
| pSE(p15a) His-NG-WALP-G spR | Spectinomycine | This study |
| pSE(p15a) His-NG-WALP-TolR-A spR | Spectinomycine | This study |
| pSE(p15a) His-NG-WALP-TolR-B spR | Spectinomycine | This study |
| pSE(p15a) His-NG-WALP-TolR-C spR | Spectinomycine | This study |
| pSE(p15a) His-NG-WALP-TolR-D spR | Spectinomycine | This study |
| pSE(p15a) His-NG-WALP-TolR-E spR | Spectinomycine | This study |
| pSE(p15a) His-NG-WALP-TolR-F spR | Spectinomycine | This study |
| pSE(p15a) His-NG-WALP-TolR-G spR | Spectinomycine | This study |
| pSE(p15a) His-NG-WALP-A kmR | Kanamycin | This study |
| pSE(p15a) His-NG-WALP-B kmR | Kanamycin | This study |
| pSE(p15a) His-NG-WALP-C kmR | Kanamycin | This study |
| pSE(p15a) His-NG-WALP-D kmR | Kanamycin | This study |
| pSE(p15a) His-NG-WALP-E kmR | Kanamycin | This study |
| pSE(p15a) His-NG-WALP-F kmR | Kanamycin | This study |
| pSE(p15a) His-NG-WALP-G kmR | Kanamycin | This study |
| pSE(p15a) His-NG-WALP-TolR-A kmR | Kanamycin | This study |
| pSE(p15a) His-NG-WALP-TolR-B kmR | Kanamycin | This study |
| pSE(p15a) His-NG-WALP-TolR-C kmR | Kanamycin | This study |
| pSE(p15a) His-NG-WALP-TolR-D kmR | Kanamycin | This study |
| pSE(p15a) His-NG-WALP-TolR-E kmR | Kanamycin | This study |
| pSE(p15a) His-NG-WALP-TolR-F kmR | Kanamycin | This study |
| pSE(p15a) His-NG-WALP-TolR-G kmR | Kanamycin | This study |
| pSE(p15a) His-NG-WALP-B-opsin spR | Spectinomycine | This study |
| pSE(p15a) His-NG-WALP-C-opsin spR | Spectinomycine | This study |
| pSE(p15a) His-NG-WALP-G-opsin spR | Spectinomycine | This study |
| pET16b His-NG-WALP-A | Ampicillin | This study |
| pET16b His-NG-WALP-B | Ampicillin | This study |
| pET16b His-NG-WALP-C | Ampicillin | This study |
| pET16b His-NG-WALP-D | Ampicillin | This study |
| pET16b His-NG-WALP-E | Ampicillin | This study |
| pET16b His-NG-WALP-F | Ampicillin | This study |
| pET16b His-NG-WALP-G | Ampicillin | This study |
